# Transformation to Neuroendocrine Phenotype in Non-Small-Cell Lung Carcinoma: A Literature Review

**DOI:** 10.3390/ijms26115096

**Published:** 2025-05-26

**Authors:** Irene Hernández de Córdoba, Xabier Mielgo-Rubio, Paloma Cejas, Jorge Palomar Ramos, Beatriz Garrido-Rubiales, Sandra Falagán Martínez, Gustavo Rubio Romero, María Morales Parga, Laura Moll Taltavull, Andrea Fernández González, Enrique Casado Sáenz, María Sereno

**Affiliations:** 1 Department of Medical Oncology, Hospital Universitario Infanta Sofía, San Sebastián de los Reyes, 28702 Madrid, Spainmariasereno75@gmail.com (M.S.); 2 Departamento de Oncología Médica, Hospital Universitario Cruces, 48903 Barakaldo, Spain; 3 Center for Functional Cancer Epigenetics, Dana-Farber Cancer Institute, Boston, MA 02215, USA; 4 Departament of Pathology, Hospital Universitario Infanta Sofía, San Sebastián de los Reyes, 28702 Madrid, Spain; 5 FIIB-HUISHHEN, Department of Medicine, Faculty of Medicine, Health and Sports, Universidad Europea de Madrid, 28670 Madrid, Spain

**Keywords:** neuroendocrine transformation, TP53, Rb1, non-small-cell lung cancer

## Abstract

Neuroendocrine transformation in non-small-cell lung cancer (NSCLC) is an uncommon but clinically significant resistance mechanism to targeted therapy, immunotherapy, and chemotherapy. This phenomenon, primarily observed in adenocarcinoma (ADC) with EGFR mutations under tyrosine kinase inhibitor (TKI) treatment, leads to histological transformation into small-cell lung cancer (SCLC), commonly associated with an aggressive clinical course and poor prognosis. Standard platinum–etoposide chemotherapy provides only transient disease control, highlighting the urgent need for improved therapeutic strategies. Early identification of transformation through biopsy, liquid biopsy, or biomarkers like neuron-specific enolase (NSE) and pro-gastrin-releasing peptide (pro-GRP) may allow for early intervention. As targeted therapies continue to develop, understanding the molecular drivers of neuroendocrine transformation is crucial for optimizing treatment. Further research into novel treatment approaches, including combination therapies with TKIs, chemotherapy, immunotherapy, and epigenetic modulators, is required to improve outcomes for these patients.

## 1. Introduction

Lung cancer (LC) is one of the most common malignant cancers worldwide, causing around 18% of total cancer mortality [1]. Two subtypes have been identified: non-small-cell lung cancer (NSCLC) (85%), also divided into adenocarcinoma (ADC), squamous-cell carcinoma (SCC), and large-cell carcinoma, and small-cell lung cancer (SCLC) (15%) [2]. Transformation from NSCLC to SCLC was first described in 2006 by Zakowski in a patient with EGFR mutation who was on Erlotinib treatment [3]. This is not an isolated phenomenon in NSCLC, and has also been seen in other tumors such as prostate cancer, where permanent hormonal blockade favors the development of more progression-aggressive histologies as resistance mechanisms [4]. In LC, neuroendocrine transformation was found to arise from NSCLC under targeted therapy, immunotherapy, and chemotherapy and most of these cases were in ADC, and very few cases happened in SCC [5]. Transformation to a small-cell phenotype is an event secondary to a persistent targeted blockade (anti-target, anti-PD-1/PD-L1), leading to the acquisition of resistance mechanisms that induce an aggressive histology, overlappable with small-cell neuroendocrine changes, with biological behaviour even more unfavorable than tumors with this de “novo” histology [6]. A full list of abbreviations used throughout this review is provided in Supplementary Table S1.

In this review, we will analyze the evidence on the main pathogenic aspects underlying neuroendocrine transformation in NSCLC as a mechanism of resistance to various target therapies and immunotherapy, as well as clinical and therapeutic features based on what has been described in the literature.

## 2. Pathogenesis of Neuroendocrine Transformation

### 2.1. Transformation of Lung Adenocarcinoma (LUAD) to Small-Cell Lung Cancer (SCLC)

**Figure 1:**
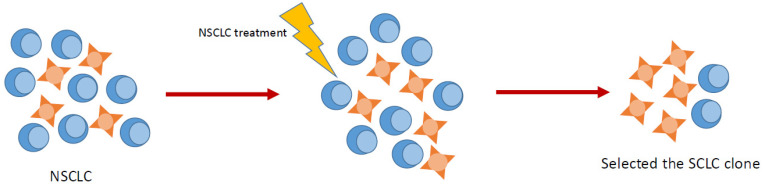
Targeted treatment against NSCLC histology selected the SCLC clone, making it dominant.

The mechanisms underlying histological transformation have not been fully elucidated and probably relate to cancer stem cells, genetic driver alterations under selective pressure, or the heterogeneity of the tumor. Regarding the pathogenic mechanisms explaining the presence of SCLC in the re-biopsy of an NSCLC progressing to targeted therapy or immunotherapy (IO), two theories were proposed (Figure 1): the first suggests that the SCLC component was present from the beginning and that targeted treatment against NSCLC histology selected the SCLC clone, making it dominant; the second proposes that the neuroendocrine (NE) histology resulted from molecular and morphological transformation of NSCLC cells secondary to continuous therapeutic blockade and the development of escape mechanisms based on mutations in *P53* and *RB* [5]. In this scenario, both before and after the diagnosis of SCLC, genetic analysis revealed *EGFR* mutation or deletion. Similar occurrences have been noted in prostate cancer, where NE differentiation emerged following chemotherapy (CT) or hormonotherapy [7]. *EGFR* mutations are rare in SCLC, yet when *EGFR* mutations matching those from the initial biopsy are discovered in subsequent biopsies, it suggests that the SCLC identified upon re-biopsy is not a new occurrence. Instead, it has likely evolved from NSCLC. Furthermore, NSCLC evolves into SCLC because they originate from the same type of cell. Research utilizing next-generation sequencing (NGS) to examine NSCLC patients who later developed SCLC found that *TP53 C176S* and *EGFR* exon 19 deletions were present in consecutive biopsy samples and varied in a parallel manner. This indicates that the transition to SCLC represents a histological shift originating from an identical cellular source [8]. As we have previously mentioned, the second hypothesis explaining the transformation of NSCLC into SCLC is based on the idea that the original tumor contained clones of cells with aggressive NE differentiation. The blocking of the non-small-cell component with CT, targeted therapy, or IO selects for clones of cells that are more resistant to these pre-existing treatments with neuroendocrine histology, which become dominant. However, given that the presence of actionable mutations common to NSCLC in SCLC histology is very rare, this theory does not explain the initially good response that these tumors would have, although over time, there would be a secondary progression due to the dominance of the more aggressive histology [9]. A study involving 4112 patients diagnosed with *EGFR/RB1/TP53*-mutant lung cancers revealed that ADC patients had a median time to SCLC transformation of up to 1.1 years after undergoing *EGFR-TKI* treatment. This finding contradicts the hypothesis that drug resistance onset and subsequent pathological manifestations of SCLC should occur sooner. Hence, it is probable that SCLC transformation from NSCLC occurs through lineage plasticity rather than the presence of dual tumors [10]. Recent experimental studies have provided further insights into the biological constraints governing histological transformation from NSCLC to SCLC. Gardner et al. (2024) demonstrated that lineage-specific oncogenic tolerance plays a critical role in permitting or restricting transformation [11]. Their work revealed that MYC, a key oncogenic driver of SCLC, is not well tolerated in alveolar type II (AT2) cells—the presumed cell-of-origin of LUAD—unless these cells undergo a reprogramming process into a basal-like intermediate state. This transition is facilitated by activation of the PI3K/AKT pathway, particularly in the context of PTEN loss. These findings highlight that transformation is not only driven by genetic events such as TP53 and RB1 inactivation, but also requires epigenetic and cellular conditions that enable neuroendocrine reprogramming. Such data support the hypothesis that histological transformation results from lineage plasticity rather than pre-existing neuroendocrine clones [11].

### 2.2. Transformation of Lung Squamous Cell Carcinoma (LUSC) to Small-Cell Lung Cancer (SCLC)

Although most cases of neuroendocrine transformation have been reported in EGFR-mutant LUAD, the transformation of lung squamous cell carcinoma (LUSC) to small-cell lung cancer (SCLC) is a recognized but relatively rare phenomenon. This histological transformation is also associated with resistance to therapies, including immunotherapy, chemotherapy, and targeted therapies. These transformations are typically associated with TP53 and RB1 alterations, similar to the transformation seen in LUAD, although the molecular pathways may differ.

Several studies have reported cases where LUSC transformed into SCLC, particularly in the context of treatment resistance. Shen et al. described two cases of LUSC transforming into SCLC during anti-PD-1 therapy, highlighting this transformation as a potential mechanism of resistance to immunotherapy [12].

## 3. Acquisition of a Neuroendocrine Phenotype as a Resistance Mechanism in Patients with Actionable Alterations 

### 3.1. EGFR

The EGFR gene is part of the human epidermal growth factor (HER) family and plays a significant role in tumor development and progression. Mutations that activate the *EGFR* gene, such as exon 19 deletions and exon 21 L858R point mutations, are found in approximately 15% of Caucasian patients and 50% of East-Asian patients with advanced NSCLC. These mutations are particularly common in female patients who have never smoked. The standard first-line treatments for NSCLC with activated *EGFR* mutations are *EGFR* tyrosine kinase inhibitors (EGFR-TKIs) like Afatinib, Erlotinib, Gefitinib, Dacomitinib, and Osimertinib, which can achieve significant disease control for a certain period [13]. The main EGFR-targeted therapies and their corresponding mutations are summarized in Supplementary Table S2.

However, resistance to *EGFR*-TKIs often develops following treatment. One of the resistance mechanisms to first- and second-generation *EGFR*-TKIs is SCLC transformation, which accounts for 14% of such cases [14].

This phenomenon has been described not only as a mechanism of resistance to TKIs but also in treatment-naive patients [15].

**Figure 2:**
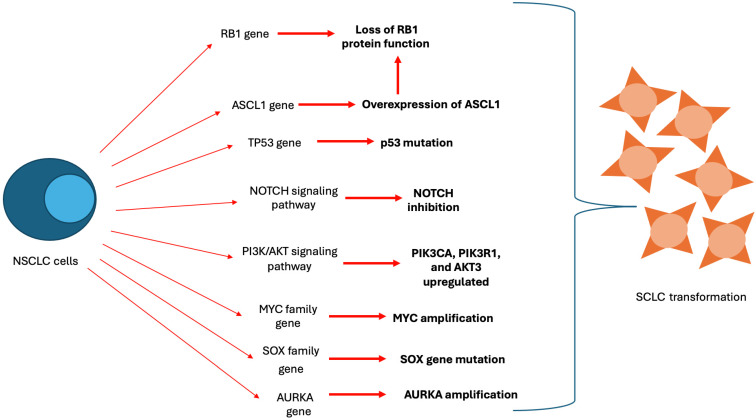
Different mechanisms of transformation to a NE phenotype described in the context of EGFR disease.

Different mechanisms of transformation to an NE phenotype have been described in the context of EGFR disease (Figure 2).

*RB1* and *TP53*: The most well-studied molecules involved in SCLC transformation are *RB1* and *TP53*, which activate mutations in the early transformation stage. *RB1* is a tumor suppressor gene that inhibits entry into the S-phase of the cell cycle, thus preventing uncontrolled proliferation. Loss of RB1 protein function drives the initiation of most cancers, including SCLC, through the disruption of cell cycle regulation; the absence of functional Rb eliminates the G1/S checkpoint, allowing cells to enter S-phase and promoting uncontrolled cellular proliferation [16].

*TP53* is an essential tumor suppressor gene, frequently mutated in various cancers, that encodes the p53 protein responsible for regulating cell division and proliferation. Mutant *TP53* promotes cancer cell proliferation and metastasis [17].

A retrospective study showed that *RB1* and *TP53* were the two most common mutations occurring in patients with SCLC that transformed from *EGFR*-mutant NSCLC, at 68% and 36%, respectively [15].

In addition, a study observed that no SCLC transformation occurred in *EGFR*-mutant lung cancers without baseline *TP53* and *RB1* alterations, as well as that patients with *EGFR/RB1/TP53*-mutant NSCLC had a higher SCLC transformation rate than those with *EGFR/TP53*-mutant *RB1*-wildtype and *EGFR*-mutant *RB1/TP53*-wildtype NSCLC, indicating that *TP53* and *RB1* alterations may enhance SCLC transformation [18].

The exact role of *RB1* and *TP53* in transformation remains uncertain. Research on prostate cancer has utilized mouse and human models with RB1 deletion, demonstrating that this deletion encourages lineage plasticity, resulting in a histological shift from prostate ADC to NE (small cell) variants. This transformation in prostate cancer relies on the increased expression of stem cell reprogramming factors like *SOX2* and *EZH2*. Consequently, *RB1* loss may also facilitate SCLC transformation by upregulating epigenetic and stem cell reprogramming factors in *EGFR*-mutant ADC [19].

While *TP53* and *RB1* mutations contribute to the transformation into SCLC, they are not sufficient to fully induce neuroendocrine (NE) transformation. A study that analyzed seven patients whose disease had transformed into SCLC found that the transformation rate for those with advanced NSCLC and *EGFR* mutations (3%) was significantly lower—by a factor of six—compared to those with *EGFR/RB1/TP53* mutations (18%). These findings suggest that the presence of *EGFR*, *RB1*, and *TP53* mutations alone does not ensure a definitive transformation into SCLC during the progression of the disease [20].

Complementary to these observations, Ding et al. reported that SCLC transformation may occur independently of EGFR mutation status and does not always require RB1 loss. In their cohort of 1474 NSCLC patients, including both EGFR-mutant and wild-type cases, 24 (7%) experienced histological transformation to SCLC. Finally, the authors identified SMAD4 as a critical regulator: its loss in TP53-deficient NSCLC cells promoted neuroendocrine differentiation via ASCL1 upregulation, even in the absence of RB1 alterations. From a mechanistic perspective, SMAD4 was shown to suppress ASCL1 by competing with MYC for MAX binding; its inactivation led to MYC-driven ASCL1 transcription. Importantly, SMAD4-deficient tumors demonstrated resistance to EGFR-TKIs and chemotherapy but enhanced sensitivity to MYC inhibition, positioning SMAD4 as both a mechanistic and therapeutic pivot in the transformation process [21].

Therefore, it is important not to overlook the role of other molecular alterations or epigenetic changes, such as specific signaling pathways, the tumor microenvironment, or hypoxia, in the transformation to SCLC.

*PI3K/AKT*: The phosphatidylinositol 3-kinase (*PI3K*)/*AKT* pathway and its signaling cascade play a role in regulating cell growth and metabolism. A study comparing differentially expressed genes in LUAD and transformed SCLC revealed that key molecules in the *PI3K/AKT* signaling pathway, such as *PIK3CA*, *PIK3R1*, and *AKT3*, were upregulated in the transformed SCLC samples [22].

*PTEN* alteration has also been found in patients with transformed SCLC from *EGFR*-mutant ADC, suggesting its similar role of increasing lineage plasticity in SCLC transformation [23].

*NOTCH*: In addition to the previously mentioned mechanisms, which are more studied in the context of NE phenotypic transformation, there are other additional molecular alterations that are also present in *EGFR*-positive ADCs with secondary resistance to TKIs.

The *NOTCH* signaling pathway is a well-conserved mechanism of intercellular communication that supports lung development in mammals by controlling cell differentiation, survival, and lineage specification [24].

Differential gene expression analyses also revealed that *DLL3* and *HES6* (genes associated with Notch signaling inhibition) are upregulated during SCLC transformation, indicating that downregulation of *NOTCH* signaling is involved in the transformation [22].

*ASCL1*: Achaete-scute homologue 1 (ASCL1) is a transcription factor from the bHLH gene family that governs neuroendocrine lineage development in multipotent stem cells. ASCL1 can also influence the *RB-P53* pathway in vivo during the development of neuroendocrine lung cancer. Studies have demonstrated that the overexpression of *ASCL1* can lead to RB inactivation through phosphorylation by promoting cyclin-dependent kinase 5 (*CDK5*) [25].

*MYC protein family*: The *MYC* protein family plays a significant role in cell reprogramming, serving as the primary transcriptional regulator that influences the activity of epigenetic control elements, and promotes a stem-like state that allows for greater plasticity. Amplification of *MYC* is frequently observed in transformed SCLC and prostate NE tumors, as well as in pre-transformed adenocarcinomas, indicating that *MYC* is a crucial factor in the early stages of transformation [26,27].

*SOX*: The *SOX* family of transcription factors is involved in reprogramming and maintaining stemlessness. Research has shown that *SOX2*, a member of the *SOX* gene family, plays a role in promoting small-cell transformation in prostate cancer with RB1/TP53 mutations and in the transformation of *EGFR*-mutant lung adenocarcinoma. However, one study discovered concurrent enrichment of *SOX17* and alterations in *EGFR/RB1/TP53* mutations in lung cancer patients, leaving *SOX2*’s direct role in the histological transformation to SCLC unclear [10,28].

*Aurora Kinase A*: Various studies have identified that amplification of the cell cycle of kinase aurora kinase A (AURKA) serves as an early marker for neuroendocrine transformation. Additionally, research has demonstrated that AURKA supports neuroendocrine differentiation in prostate and lung adenocarcinomas by interacting with *N-MYC*. This suggests that AURKA could potentially drive the transformation to SCLC [29,30].

*KRAS*: In addition, studies have noted lower *KRAS* mutations in LUAD transformed to SCLC, indicating that *KRAS*-mutant LUAD is less likely to undergo NE transformation [22].

### 3.2. ALK

The specific mechanism by which ALK fusion-positive cells acquire a neuroendocrine (NE) phenotype is not well established. Similarly to EGFR patients, the loss of *RB1* and *TP53* may be responsible for the acquisition of this more aggressive phenotype [31].

The inactivation of NOTCH1 at diagnosis has been linked by some authors to an increased risk of transformation to SCLC after TKI treatment [32].

First-generation (Crizotinib) and second-generation (Ceritinib, Alectinib, Brigatinib) TKIs are approved as first-line treatments for advanced *ALK*-positive NSCLC patients due to their proven survival benefits over chemotherapy. Alectinib and Brigatinib, both second-generation TKIs, have now replaced Crizotinib as the standard of care, thanks to their improved safety profiles and enhanced effectiveness in targeting intracranial disease.

Lorlatinib, a third-generation ALK TKI, is now recommended as the second-line treatment for ALK-positive NSCLC that progresses after treatment with Alectinib, Ceritinib, or Crizotinib [33].

However, the probability of selected *ALK* mutations driving acquired resistance increases after administering last-generation *ALK* TKIs, and approximately 50% of cases develop resistance to second-generation *ALK* TKIs. Among them, the most common is G1202R (35–60%) with Lorlatinib being the only *ALK* TKI that seems to be active against this point mutation.

Off-target mechanisms of resistance include bypass signaling (such as *EGFR*, *MET*, *c-KIT*, *SRC*, *RAS/MAPK*, and Src-homology 2 domain-containing phosphatase *2-SHP2* mutations) [34].

Among the treatment options with *ALK* inhibitor TKIs, various authors, such as Fujita et al., have demonstrated that the probability of transformation is higher with more potent inhibitors, as patients are subjected to greater inhibitory pressure [35].

There is no standard treatment strategy for transformed SCLC patients after *ALK*-TKI treatment. Cisplatin–irinotecan, the standard therapy for SCLC in Japan, or cisplatin–etoposide, the standard therapy in America and Europe, may be options with a continuous partial response in the primary lesion and in the other lesions [36]. The combination of TKI and chemotherapy could be a reasonable option in *ALK* patients [37].

Experience with IO alone or in combination with chemotherapy (CT) is limited in transformed-ALK patients, and to date, there are no studies evaluating the role of CT and IO in patients with SCLC transformed from adenocarcinoma with *ALK* translocation.

### 3.3. ROS-1

*ROS1* rearrangements occur in 1–2% of advanced NSCLC. Crizotinib has been approved by the food and drug administration (FDA) and European Medicines Agency (EMA) as a front-line therapy in *ROS1*-rearranged advanced NSCLC according to the PROFILE 1001 study [38]. In the same way as occurs with other targeted therapies, the development of resistance mechanisms favors a lack of inhibition efficacy [39]. Recently, new therapeutic options have emerged that are more potent (Entrectinib, Lorlatinib, Repotrectinib) and have more intracerebral activity, a condition especially interesting for these diseases, which have a high tendency to invade the central nervous system [40,41]. The main resistance mechanisms to anti-ROS-1 therapies are secondary mutations, detected in around 50–60% of patients treated with Crizotinib (*G2032R*, *D2033N*, *L2026M*, *S1986F/Y*, *K1991E*) [42], some of which are sensitive to Lorlatinib (*S1986F* and *K1991E*), whereas Repotrectinib has shown activity against *G2032R* [43,44]. Off-target mechanisms of resistance to *ROS1* include the activation of other signaling pathways such as *EGFR*, *MET*, *HER2*, *KRAS*, *KIT*, *BRAF*, and *MEK* [39].

However, there is very little evidence regarding the transformation of these types of tumors to an aggressive phenotype. Filleti M et al. published a case report of a 45-year-old patient with a cytological diagnosis of large-cell neuroendocrine lung carcinoma who, after starting platinum–etoposide-based chemotherapy, developed cerebral progression. Genomic sequencing was performed, revealing a *ROS1* rearrangement, and targeted therapy with Crizotinib was initiated with a very good response. Since NGS was not performed at the time of diagnosis, it is likely that the translocation was present from the beginning and, therefore, that the response to chemotherapy was worse than the response to the TKI. Another question pertains to whether the neuroendocrine phenotype could have become more aggressive after chemotherapy or as a natural evolution of the initial histology [45].

Other transformations into aggressive subtypes, such as sarcomatoid, in patients with *ROS-1* translocations have also been described. Ko HJ et al. reported a case of a 43-year-old non-smoker female who was diagnosed with right upper lung adenocarcinoma with pericardial effusion, spine metastasis, and solitary brain metastasis and *ROS-1* rearrangement confirmed by FISH. She was initially treated with cisplatin and Pemetrexed and shifted to Crizotinib due to the rapid progression of malignant pericardial and pleural effusion after 1 cycle. After 10 months, she developed a pleural progression and lymphangitic carcinomatosis secondary to a pulmonary sarcomatoid carcinoma, maintaining *ROS-1* translocation, and with a good response to Lorlatinib [46].

### 3.4. K-RAS

*KRAS* mutations are the most common oncogenic drivers in NSCLC (20–25%), and among them, *KRAS-G12C* is the most frequent (50%) [47]. Recently, we developed drugs targeted at this type of mutation, approved for progression following CT- and IO-based regimens, with interesting response rates and PFS (progression-free survival) and better tolerance than to Docetaxel, the standard therapy in the context of second-line treatment and progression to platinum +/− anti-PD-1/PD-L1. Sotorasib (CodeBreak 100, 200) and Adagrasib (KRYSTAL-1, -12) are the two most advanced KRAS G12C inhibitors for this type of patient; however, there is a percentage of patients who do not respond to this medication [48].

Most resistance mechanisms to these *KRAS G12C* inhibitors are based on *KRAS* mutations or the activation of different signaling pathways: *SOS Ras/Rac* guanine nucleotide exchange factor 1 (*SOS1*), aurora kinase A (*AURKA*), or EGFR/fibroblast growth factor receptor *(FGFR)/PI3K* [49,50,51].

To prevent the development of resistance, there are an increasing number of studies aimed at reversing resistance to *KRAS* inhibitors with other inhibitors targeting resistance pathways: *EGFR* inhibitors (Panitumumab, Cetuximab, Afatinib), *CDK4/6* inhibitors (Palbociclib), *MEK* inhibitors (Trametinib + Panitumumab), and *mTOR* inhibitors (Everolimus); however, these studies are still in very preliminary stages [52].

After reviewing the literature, no cases have been described of NSCLC patients with *KRAS* mutations presenting a transformation to small-cell histology. One of the reasons is the absence of re-biopsies in the context of metastatic *KRAS* disease, as there are no approved treatments based on these findings beyond clinical trials. It is likely that with the availability and universal adoption of sequencing techniques, performing re-biopsies upon progression will become a more generalized practice, and thus, as occurs with other targets, we may be able to detect cases of phenotypic transformation.

### 3.5. RET

*RET* fusions are found in 1–2% of NSCLC cases, and the most common patterns of fusions are kinesin family member 5B *(KIF5B)-RET* (70–90%) and coiled-coil domain-containing 6 *(CCDC6)-RET* (10–25%) [53]. There are various targeted treatments for this alteration, ranging from multitarget TKIs (Cabozantinib, Lenvatinib, Vandetanib, and Ponatinib), with modest activity and an improbable safety profile, to highly selective *RET* fusion TKIs, such as Pralsetinib and Selpercatinib, which show high efficacy and an excellent safety profile [54,55].

Based on data from studies with these new inhibitors, the use of these selective TKIs has been approved as first-line treatments (FDA) and upon progression to CT and IO (EMA) [54,56].

Pishdad R et al. published a case report of a patient with a history of Hodgkin’s lymphoma treated with RT who later developed a calcitonin-rich NE carcinoma with a *RET* translocation, showing an excellent response to Selpercatinib and eventual resistance to it, associated with an activating mutation involving the *MEK1* protein (*MAP2K1 p. E102-I103 del*) that led to relapse and disease progression [57].

However, similarly to what happens with other mutations, despite the initial response to these treatments, the patient eventually developed resistance through various mechanisms. Among the main resistance mechanisms to *RET* inhibitors are *MET* amplifications, for which multikinase inhibitors like Cabozantinib, with both anti-*MET* and anti-*RET* activity, are used in these cases [55].

On-target mechanisms include secondary *RET* mutations (e.g., *G810C*, *G810S*, *V804*) responsible for resistance to selective inhibitors. Phenotypic transformation in patients with *RET* alterations is a rarely described event in the literature, both in the context of *RET* translocations or fusions and in cases of exon 14 skipping mutations.

### 3.6. MET

There are various *MET* alterations in lung cancer, including amplifications (1–3%) or fusions and point mutations, such as *MET* exon 14 skipping mutation (3–4%). The main selective treatments approved for managing patients with these alterations are Tepotinib and Capmatinib for those with exon 14 skipping mutations, and previously, Crizotinib as a non-selective treatment, since it also has activity against *ALK*, making it the treatment of choice in this pathology for several years [58].

Among the resistance mechanisms to MET inhibitors are on-target resistance mechanisms based on other mutations, such as type I Crizotinib and Capmatinib resistance (*D1228*, *Y1230*, *H1094*, *G1163*, and *L1195*) or type II resistance to glesatinib [59].

We have not found published cases of transformation to small-cell histology in patients with *MET* mutation or amplification in LC. One possible explanation is the lack of routine biopsies after progression in this context, as the treatments for resistance, outside of clinical trials, are mostly chemotherapy, especially if significant clinical worsening is observed.

### 3.7. BRAF

BRAF mutations in non-small-cell lung cancer account for around 5% of adenocarcinomas, which include two types of sub-mutations: V600 and non-V600. Several studies have demonstrated high efficacy of selective inhibitors in combined anti-*BRAF-MEK* therapy, such as Dabrafenib combined with Trametinib, although this activity is limited to V600 mutations [60].

As we have mentioned in previous paragraphs, the initial activity of these treatments is associated with the development of resistance mechanisms that include mutations affecting the *RAS-RAF-MEK* pathway, such as *KRAS* mutations (*Q61R, G12V)/NRAS (Q61R, Q61K)*; the activation of *PI3K-AKT* and the *MEK1 K57N* and *PTEN N329fs* pathways; pathway-independent alterations (*NADP (+) 1 (IDH1)*); *U2* small nuclear RNA auxiliary factor 1 (*U2AF1*); and catenin beta 1 (*CTNNB1*) alterations [61,62].

Although we have not found cases of transformation to small-cell histology in this subgroup of patients treated with inhibitors, *BRAF* mutations have been described as a resistance mechanism in patients with *EGFR* mutations treated with inhibitors [63].

On the other hand, Mariniello A, et al. described transformation to a squamous histology phenotype in a patient with an initial adenocarcinoma carrying a non-V600 *BRAF* mutation under immunotherapy (anti-PD-L1). After early hyperprogression, the biopsy confirmed the transformation to a squamous phenotype [64].

### 3.8. NTRK

*NTRK* fusions are very rare alterations in the field of LC, being more common in other pathologies such as thyroid cancer or pediatric tumors [65]. Various inhibitors have been developed, such as Larotrectinib and Entrectinib, with potent activity demonstrated in different phase I-II studies designed for various pathologies [66].

Similarly, emerging resistance mechanisms include secondary mutations or new pathways (including BRAF-V600E, KRAS-G12D, and MET amplification), but again, small-cell transformation is a fairly rare phenomenon in this context, and to date, histological transformations have not been described among the resistance mechanisms in this type of tumor.

## 4. Acquisition of a Neuroendocrine Phenotype as a Mechanism of Resistance to Immunotherapy

The immune checkpoint inhibitors PD-1 and PD-L1 (Nivolumab, Pembrolizumab, and Atezolizumab) are part of the treatment for NSCLC, both as monotherapy and in combination with CT in early and advanced stages. However, the mechanisms of resistance to IO have not been elucidated.

Recently, with the widespread use of these treatments, NE transformations have been described in several published cases [67].

In these cases, the time to transformation was quite variable (ranging from 2 weeks to nearly 3 years), and the response ranged from partial response to progression. The molecular analysis of biopsies with NE transformation showed mutations in *P53/RB*, as has been described in several studies analyzing this event, especially in *EGFR* patients. Bar et al., in a series of 249 patients treated with ICI, found two cases of small-cell transformation among the eight cases biopsied at progression. Both patients showed poor outcomes despite platinum–etoposide treatment.

The authors conclude by stating the importance of performing serial biopsies in cases of progression to IO or paradoxical responses [68].

This phenomenon has also been described in other tumors, such as colon cancer. Du F et al. published the case of a patient with colon adenocarcinoma undergoing treatment with anti-angiogenic and anti-PD-L1 therapy, who developed a large-cell NE carcinoma transformation upon progression, detected in a re-biopsy performed due to a discordant response to the treatment. The mutational study of the transformed histology biopsy showed the same alterations as the original tumor. After confirming this histological change, there was significant worsening of the patient’s clinical course [69].

In addition to the transformation associated with ICI monotherapy, Xin G et al. reported the case of a patient initially diagnosed with stage II esophageal squamous cell carcinoma who underwent radical surgery after three cycles of neoadjuvant (NA) therapy based on a combination of CT-IO: cisplatin, Albumin-bound Paclitaxel, and ICIs. In the pathological specimen after surgery, immunohistochemical staining confirmed the absence of an SCC component and the presence of an NEC component, with negativity for CK5/6 and tumor protein p40, but positive expression of tumor protein p53, pan-cytokeratin, synaptophysin, and CD56. The outcome was favorable, and no complications were described [70].

Sehgal et al. published a case of a patient who had a small-cell transformation of stage IV poorly differentiated squamous cell carcinoma of the lung after prolonged Nivolumab second-line monotherapy for 21 months. After sustained stabilization, the patient experienced clear progression with a bulky mediastinal mass, which was biopsied and revealed transformation into small-cell carcinoma. Tumor genomic profiling performed at initial diagnosis and following disease progression on Nivolumab showed nearly identical results. The absence of neuroendocrine features on the initial biopsy, the protracted response to Nivolumab monotherapy, and the nearly identical genomic profiles of both tumors supported the diagnosis of histological transformation in this patient. Treatment with carboplatin–etoposide led to a near-complete response, although it only lasted 10 months, with chest consolidation treated with radiation and Nivolumab. However, after this last treatment, the patient experienced progressive worsening, and 14 months after the detection of neuroendocrine transformation, the patient’s condition deteriorated further [71].

The same authors published a systematic review on neuroendocrine transformation after anti-PD-1/PD-L1 treatment in non-small-cell lung cancer (NSCLC) patients. From five articles, nine patients were identified. All of these patients were undergoing treatment with immune checkpoint inhibitors (ICIs) at the time of small-cell lung cancer (SCLC) detection; seven (77.8%) were on Nivolumab and two (22.22%) were on Pembrolizumab monotherapy. Five (55.6%) of the patients were male, with a median age of 68 years (ranging from 65 to 75 years). All eight (100%) patients had a documented smoking history had a history of tobacco use. The median number of treatments before starting IO was 1 (ranging from 0 to 3). Every patient (100%) had received CT before switching to either second-line or IO maintenance. Following the detection of small-cell transformation, seven (77.8%) patients received carboplatin-etoposide as the next immediate line of therapy. For the eight patients with available survival data, the median survival time from the detection of small-cell transformation was 13.0 months (95% CI: 2.0 to 16.0 months), which is comparable to the 10.9 months (95% CI: 8.0 to 13.7 months) previously reported in cases of transformed EGFR-mutant lung ADC treated with TKIs [71].

No consensus guidelines exist on how to define NSCLC-to-small-cell transformation and distinguish it from new primary SCLC.

The real-world frequency of histological transformation with IO is still uncertain. It is likely under-recognized and under-reported due to the infrequency of tumor re-biopsy in advanced NSCLC patients who are being treated with sequential chemotherapies and/or ICI. As previously mentioned, Bar et al. conducted a study on biopsies at the time of NSCLC progression on ICIs at a single institution. They reported a small-cell transformation rate of 25% in eight NSCLC patients with available pre-progression and post-progression tissue biopsies (though two post-progression biopsies did not show any tumor cells). In contrast, Gettinger et al. found no histological changes in lung cancer (0%) when evaluating 23 NSCLC cases (all containing tumor cells), from a single institution, with acquired resistance to anti-PD-1 drugs [72]. Both studies are constrained by small sample sizes, highlighting the need for prospective research on this potential phenomenon. The genomic and epigenetic changes underlying this mechanism of therapeutic resistance require more comprehensive investigation. The real-world incidence of histological transformation with IO is still uncertain and is likely under-recognized and under-reported due to the rarity of tumor re-biopsy in advanced NSCLC patients treated with sequential chemotherapies and/or IO. In the meantime, we recommend considering tissue biopsies at the time of NSCLC progression under IO, similar to the approach with TKIs, if it is safe and feasible for the patient.

## 5. Clinical Features Associated with a Higher Risk of Neuroendocrine Transformation

No clear clinical features have been identified that are associated with an increased risk of neuroendocrine transformation. Some case reports have also evaluated the relationship between clinical characteristics and the occurrence of SCLC transformation. For instance, female gender is related to a later occurrence of transformation, while smoking is associated with the opposite trend based on univariate analysis [73].

In *EGFR* patients, a poor response to *EGFR*-TKIs and rapid tumor progression suggest SCLC transformation. However, these conclusions are only based on case observations. Further studies are needed to assess whether they are related to transformed SCLC and to determine their effects on transformation [74].

## 6. Diagnosis

One of the most significant challenges of neuroendocrine transformation in patients with driver mutations is early diagnosis. It is not always possible to diagnose due to the absence of accessible lesions or the lack of confirmation of the neuroendocrine phenotype when definitive treatments like SBRT ablation are performed, especially in cases of oligo-progression.

Biopsy is the most reliable method to diagnose SCLC transformation. Performing a repeated biopsy when disease progresses can effectively enable the early identification of whether a transformation occurred. Repeat biopsy of primary sites, lymph nodes, or possible metastatic sites can enhance the diagnosis and early detection of transformed SCLC. However, because surgical resection cannot be conducted on all patients, a biopsy can only collect limited tumor tissue in these cases; liquid biopsy (LB) could be an option to overcome this limitation.

Liquid biopsy (LB) is a minimally invasive method that can capture tumor-derived elements, such as circulating tumor DNA (ct DNA) and circulating tumor cells (CTCs). Certain molecular biomarkers linked to SCLC, like *RB1*, *TP53*, and others, can be used to confirm a transformation diagnosis.

Massive sequencing techniques (NGS) and droplet digital PCR (dd PCR) performed on tissue or ctDNA, when histological findings are not very clear due to limited cellular material, can help us confirm this transformation, especially when mutations in TP53 and RB1, which were not present in the initial molecular analysis, are detected.

A study noted a rapid rise in the allelic fraction of *TP53*, *RB1*, and *PIK3CA* mutations both before and during histologic transformation, suggesting that these mutations may serve as predictors for SCLC transformation [10].

In addition to biopsies, tracking gradual increases in blood levels of neuron-specific enolase (NSE) or pro-gastrin-releasing peptide (pro-GRP) can assist in identifying SCLC clones before they become apparent, serving as a supplementary diagnostic tool for SCLC transformation. NSE levels show a significant rise after transformation to SCLC. Similarly, pro-GRP has been employed as a tumor biomarker to forecast early histologic changes from NSCLC to SCLC. Regular monitoring of serum NSE and pro-GRP levels is crucial for the early detection of SCLC transformation prior to performing invasive biopsies. Nonetheless, the sensitivity and accuracy of this approach still need to be thoroughly evaluated [6,75]. 

The early detection of SCLC transformation can help change treatment plans as soon as possible, thus preventing further deterioration of tumors and prolonging the survival time of patients. Currently, there is no specific diagnostic method for detecting SCLC transformation. However, some unusual events occurring during the treatment can indicate a transformation. For instance, the primary lesion size is increased during *ALK* inhibitor treatment, while other metastatic lesions are significantly reduced. The enlargement of mediastinal nodes and pulmonary nodules indicates the development of resistance to TKI or previous treatments. Therefore, observing the primary tumor and metastasis during the treatment process can indicate tumor resistance, which may result in SCLC transformation [35].

In addition, some studies use NGS to detect gene variations in homogeneous blood samples in ctDNA, as a supplement to re-biopsy analysis, to avoid the inaccuracy associated with local biopsy samples [36].

## 7. Prognosis

The prognosis for patients with SCLC that has developed from EGFR-mutant NSCLC and other targeted LCs is poor. A case series indicated that patients with NSCLC harboring mutations in the EGFR, RB1, and TP53 genes have a median survival of 9.5 months following TKI therapy. After transformation to SCLC, the median survival increases to 29.1 months, but this outcome remains unsatisfactory [10]. Roca et al., in a systematic review, analyzed 17 patients with LUAD and 16 patients who underwent first- and second-generation TKIs, and they showed that the median overall survival (OS) after transformation to SCLC was six months, while the patient receiving Osimertinib survived for only two months. Perhaps a blockade by more potent *EGFR* inhibitors (third generation) would promote a more aggressive transformation phenotype capable of overcoming resistance to the blockade [73].

Marcoux et al., in a series with 58 NSCLC patients with *EGFR* mutations, found that the median time to transformation, median overall survival after diagnosis, and median survival after SCLC transformation were 17.8, 31.5, and 10.9 months, respectively [15].

Some studies have also identified that transformed SCLC has a significantly higher copy number variation (CNV) burden than initial LUAD in patients with enhanced C>A transversions and reduced C>T transitions. Therefore, early detection of CNV burden could predict the prognosis of CNV burden, with a more aggressive disease occurring in patients with more copies, a more aggressive phenotype, and a worse prognosis [76].

## 8. Therapeutic Options

The standard treatment for patients who have undergone phenotypic transformation currently involves a combination of etoposide and cisplatin/carboplatin-based CT. However, to improve the outcomes for these patients, and based on the biological characteristics of these tumors compared to “de novo” SCLC, new therapeutic strategies have been developed, which will be detailed below.

### 8.1. Chemotherapy

As previously mentioned, chemotherapy is a common treatment for transformed SCLC, particularly the combination of platinum and etoposide (PE). Transformed SCLC cases often show increased sensitivity to chemotherapy and a favorable response to initial treatment with etoposide and cisplatin [77].

In a retrospective study treating transformed SCLC with PE, 46 patients showed a clinical response rate of 54% at the time of transformation. However, the median progression-free survival was only 3.4 months (ranging from 2.4 to 5.4 months) [15].

Patients with transformed SCLC can achieve a similar objective response rate to CT to those with classical SCLC, around 80%. However, the long-term outlook for patients with transformed SCLC is less favorable compared to those with classical SCLC [78].

In addition to platinum-based combinations, it is worth noting that the clinical response rate to taxanes is relatively high in EGFR-mutant-transformed SCLC. For example, a retrospective study of 21 patients who were treated with taxanes later in their course showed that seven patients had a 71% response rate to Paclitaxel or Nab-paclitaxel, whereas the response rate to Docetaxel was zero [15].

### 8.2. Chemotherapy–Tyrosine Kinase Inhibitors

To increase the benefit of CT in this context, and taking advantage of the coexistence of histological changes and actionable mutations in cell clones that are sensitive to targeted inhibition, various authors have analyzed the effect of combining CT and tyrosine kinase inhibitors (TKI). The strongest evidence comes from patients with transformed ADC harboring EGFR mutations that are resistant to TKI. *EGFR*-TKIs combined with CT have also been suggested in the treatment of transformed SCLC, which delay the time to drug resistance and SCLC transformation. Some authors have reported that combination therapy might be effective in treating SCLC transformation, likely due to the extensive elimination of *EGFR*-mutant LUAD cells after the use of *EGFR*-TKIs and the suppression of EP on *EGFR/TP53/RB1* triple mutant sub-clones that resist EGFR-TKI treatment. However, there is a lack of randomized studies comparing the two strategies (CT plus TKI vs. CT alone) [10].

### 8.3. BCL-2 Inhibitors

BCL-2 family proteins regulate mitochondrial apoptosis and are anti-apoptotic proteins. The overexpression of these anti-apoptotic proteins has also been described as a molecular alteration present in the neuroendocrine transformed phenotype in LC and other tumors [79].

Therefore, as various authors have demonstrated, earlier studies have indicated that SCLC can be treated by targeting *BCL-2*. Consequently, *ABT-263*, an oral inhibitor of *BCL-2* family proteins, has been developed. *ABT-263* works by binding directly to *BCL-2*, which prevents its interaction with Bim (a pro-apoptotic *BH3*-only protein from the Bcl-2 family), thereby inducing apoptosis through Bim [80].

Niederst et al. found that SCLC-transformed cell lines respond better to *ABT-263* than *EGFR*-TKI-resistant NSCLC cell lines with the T790M resistance mutation. Additionally, *ABT-263* can greatly increase the apoptotic response in EGFR-mutant-transformed SCLC [20].

However, randomized studies are needed to confirm the efficacy of this new therapeutic strategy, not only in the context of EGFR disease, but also in neuroendocrine transformations of patients with other types of mutations, on which there are no published studies to date.

### 8.4. Targeting RB1 Loss

Several potential agents targeting *RB1* loss in transformed SCLC cells have been suggested to treat transformed SCLC. *RB1* loss placed stress on the DNA replication machinery, thus promoting sensitivity to checkpoint kinase (CHK), *CDC25* phosphatase, polo-like kinase (PLK), and AURK inhibitors. Thus, CHK and PLK inhibitors can efficiently treat transformed SCLC characterized by total RB1 loss by targeting DNA damage checkpoints [81,82].

The loss of *RB1* also results in a reliance on *AURKA*, making *AURKA* inhibitors a viable option to target RB1-deficient SCLC cells in transformed SCLC. Additionally, third-generation *EGFR*-TKIs can activate *AURKA* in *RB1*-proficient cells, preventing EGFR-TKI-induced apoptosis and thereby fostering drug resistance. Hence, AURKA inhibitors can also help delay or prevent SCLC transformation in EGFR-mutant NSCLC by reducing resistance to *EGFR*-TKIs [83].

### 8.5. Immunotherapy

In de novo SCLC, there are several studies evaluating the role of IO as a monotherapy or in combination with CT, showing modest benefits in terms of PFS and OS. However, in the context of neuroendocrine transformation following targeted therapy in patients with actionable mutations, the evidence regarding the benefit of IT in this setting is quite limited, mainly in pretreated patients with *EGFR* mutations [84].

The available evidence comes from case series or case reports. For instance, Marcoux et al. reported that no patients had an effective response after immunotherapy in a retrospective study [15].

In a different case study, a patient with transformed SCLC was treated with Nivolumab, but the disease continued to progress. Furthermore, a retrospective re-biopsy conducted later revealed no expression of programmed death ligand 1 (PD-L1) [85].

Similarly, Nishikawa et al. reported another case with EGFR mutation with negative PD-L1 expression treated with Nivolumab after transformation to SCLC, with limited efficacy, and it significantly downregulated PD-L1 compared with its initial expression in the same tumors, suggesting that *EGFR*-TKI treatment before transformation may downregulate PD-L1 [86].

The weak response of transformed SCLC to PD-L1 inhibitors is comparable to that of EGFR-mutant NSCLC to immunotherapy, suggesting similar biological behavior between transformed SCLC and *EGFR*-mutant NSCLC. More research is required before concluding that immune checkpoint inhibition is unnecessary in transformed SCLC patients. Additionally, combining immunotherapy with other treatments, such as chemotherapy and antiangiogenic therapy, could be a future direction for SCLC treatment, as it has shown potential in the treatment of *EGFR*-mutant adenocarcinoma and classical SCLC [87].

### 8.6. Anti-Angiogenic Therapy

Anti-angiogenic agents have also been analyzed as part of the treatment for patients with transformed tumors, mostly in case series. For example, Ding et al. conducted a study with *EGFR*-mutant patients pretreated with TKIs, who received anti-angiogenic agents in combination with first- or second-line CT, showing that patients receiving Anlotinib achieved a longer OS compared to those who did not receive anti-angiogenic agents [88].

However, additional clinical trials are required to evaluate the effectiveness of anti-angiogenic therapies.

## 9. Conclusions

Transformed small-cell lung cancer represents a distinct subtype of small-cell lung cancer, differing from both classical SCLC and NSCLC. This transformation is a mechanism of acquired resistance to treatments like CT, targeted therapy, IO, and RT, which worsens patient prognosis. Mutations in the *RB1* and *TP53* genes are commonly found in transformed SCLC at the molecular level, suggesting that alterations in both genes may play a role in this transformation. Due to its association with poor outcomes, early detection of SCLC transformation is crucial. Monitoring through imaging and conducting timely biopsies when changes are detected can aid in early diagnosis.

For patients with transformed SCLC and specific targeted mutations, CT, either alone or combined with tyrosine kinase inhibitors, is currently the first-line treatment and has shown effectiveness in clinical practice. Additionally, several drugs targeting epigenetic changes, such as *BCL-2* inhibitors, *CHK* inhibitors, *PLK* inhibitors, *AURK* inhibitors, and *EZH2* inhibitors, are being considered for treating transformed SCLC, based on its lineage plasticity during the transformation from *EGFR*-mutant NSCLC. Further investigation into tumor signaling pathways during this transformation could uncover new mechanisms, leading to the development of novel biomarkers and therapies.

## Supplementary Materials

The following supporting information can be downloaded at: https://www.mdpi.com/article/10.3390/ijms26115096/s1.

## References

[1] Sung H., Ferlay J., Siegel R.L., Laversanne M., Soerjomataram I., Jemal A., Bray F. (2021). Global cancer statistics 2020: GLOBOCAN estimates of incidence and mortality worldwide for 36 cancers in 185 countries. CA Cancer J. Clin..

[2] Pikor L.A., Ramnarine V.R., Lam S., Lam W.L. (2013). Genetic alterations defining NSCLC subtypes and their therapeutic implications. Lung Cancer.

[3] Zakowski M.F., Ladanyi M., Kris M.G. (2006). EGFR mutations in small-cell lung cancers in patients who have never smoked. N. Engl. J. Med..

[4] Chen X., Shao Y., Wei W., Zhu S., Li Y., Chen Y., Li H., Tian H., Sun G., Niu Y. (2022). Correction: Androgen deprivation restores ARHGEF2 to promote neuroendocrine differentiation of prostate cancer. Cell Death Dis..

[5] Ferrer L., Levra M.G., Brevet M., Antoine M., Mazieres J., Rossi G., Chiari R., Westeel V., Poudenx M., Letreut J. (2019). A brief report of transformation from NSCLC to SCLC: Molecular and therapeutic characteristics. J. Thorac. Oncol..

[6] Norkowski E., Ghigna M.-R., Lacroix L., Le Chevalier T., Fadel É., Dartevelle P., Dorfmuller P., de Montpréville V.T. (2013). Small-cell carcinoma in the setting of pulmonary adenocarcinoma: New insights in the era of molecular pathology. J. Thorac. Oncol..

[7] Walker G.E., Antoniono R.J., Ross H.J., Paisley T.E., Oh Y. (2006). Neuroendocrine-like differentiation of non-small cell lung carcinoma cells: Regulation by cAMP and the interaction of mac25/IGFBP-rP1 and 25.1. Oncogene.

[8] Zhou Y.-Z., Jin J., Tian P.-W., Li W.-M. (2018). Application of the next-generation sequencing technology to reveal mechanism of small cell lung cancer transformation from adenocarcinoma. Chin. Med. J..

[9] Park K.-S., Liang M.-C., Raiser D.M., Zamponi R., Roach R.R., Curtis S.J., Walton Z., Schaffer B.E., Roake C.M., Zmoos A.-F. (2011). Characterization of the cell of origin for small cell lung cancer. Cell Cycle.

[10] Offin M., Chan J.M., Tenet M., Rizvi H.A., Shen R., Riely G.J., Rekhtman N., Daneshbod Y., Quintanal-Villalonga A., Penson A. (2019). Concurrent RB1 and TP53 alterations define a subset of EGFR-mutant lung cancers at risk for histologic transformation and inferior clinical outcomes. J. Thorac. Oncol..

[11] Gardner E.E., Earlie E.M., Li K., Thomas J., Hubisz M.J., Stein B.D., Zhang C., Cantley L.C., Laughney A.M., Varmus H. (2024). Lineage-specific intolerance to oncogenic drivers restricts histological transformation. Science.

[12] Shen Q., Qu J., Sheng L., Gao Q., Zhou J. (2021). Case report: Transformation from non-small cell lung cancer to small cell lung cancer during anti-PD-1 therapy: A report of two cases. Front. Oncol..

[13] Yoneda K., Imanishi N., Ichiki Y., Tanaka F. (2019). Treatment of Non-small Cell Lung Cancer with EGFR-mutations. J. UOEH.

[14] He J., Huang Z., Han L., Gong Y., Xie C. (2021). Mechanisms and management of 3rd-generation EGFR-TKI resistance in advanced non-small cell lung cancer (Review). Int. J. Oncol..

[15] Marcoux N., Gettinger S.N., O’kane G., Arbour K.C., Neal J.W., Husain H., Evans T.L., Brahmer J.R., Muzikansky A., Bonomi P.D. (2019). EGFR-Mutant Adenocarcinomas That Transform to Small-Cell Lung Cancer and Other Neuroendocrine Carcinomas: Clinical Outcomes. J. Clin. Oncol..

[16] Febres-Aldana C.A., Chang J.C., Ptashkin R., Wang Y., Gedvilaite E., Baine M.K., Travis W.D., Ventura K., Bodd F., Yu H.A. (2022). Rb Tumor Suppressor in Small Cell Lung Cancer: Combined Genomic and IHC Analysis with a Description of a Distinct Rb-Proficient Subset. Clin. Cancer Res..

[17] Giaccone G., He Y. (2023). Current knowledge of small cell lung cancer transformation from non-small cell lung cancer. Semin. Cancer Biol..

[18] Sivakumar S., Moore J.A., Montesion M., Sharaf R., Lin D.I., Colón C.I., Fleishmann Z., Ebot E.M., Newberg J.Y., Mills J.M. (2023). Integrative Analysis of a Large Real-World Cohort of Small Cell Lung Cancer Identifies Distinct Genetic Subtypes and Insights into Histologic Transformation. Cancer Discov..

[19] Shaurova T., Zhang L., Goodrich D.W., Hershberger P.A. (2020). Understanding lineage plasticity as a path to targeted therapy failure in EGFR-mutant non-small cell lung cancer. Front. Genet..

[20] Niederst M.J., Sequist L.V., Poirier J.T., Mermel C.H., Lockerman E.L., Garcia A.R., Katayama R., Costa C., Ross K.N., Moran T. (2015). RB loss in resistant EGFR mutant lung adenocarcinomas that transform to small-cell lung cancer. Nat. Commun..

[21] Ding X., Shi M.-X., Liu D., Cao J.-X., Zhang K.-X., Zhang R.-D., Zhang L.-P., Ai K.-X., Su B., Zhang J. (2024). Transformation to small cell lung cancer is irrespective of EGFR and accelerated by SMAD4-mediated ASCL1 transcription independently of RB1 in non-small cell lung cancer. Cell Commun. Signal..

[22] Quintanal-Villalonga A., Taniguchi H., Zhan Y.A., Hasan M.M., Chavan S.S., Meng F., Uddin F., Manoj P., Donoghue M.T., Won H.H. (2021). Multiomic analysis of lung tumors defines pathways activated in neuroendocrine transformation. Cancer Discov..

[23] Tsui D.W.Y., Murtaza M., Wong A.S.C., Rueda O.M., Smith C.G., Chandrananda D., Soo R.A., Lim H.L., Goh B.C., Caldas C. (2018). Dynamics of multiple resistance mechanisms in plasma DNA during EGFR-targeted therapies in non-small cell lung cancer. EMBO Mol. Med..

[24] Sharif A., Shaji A., Chammaa M., Pawlik E., Fernandez-Valdivia R. (2020). Notch transduction in non-small cell lung cancer. Int. J. Mol. Sci..

[25] Meder L., König K., Ozretić L., Schultheis A.M., Ueckeroth F., Ade C.P., Albus K., Boehm D., Rommerscheidt-Fuss U., Florin A. (2016). NOTCH, ASCL1, p53 and RB alterations define an alternative pathway driving neuroendocrine and small cell lung carcinomas. Int. J. Cancer.

[26] Khan P., Siddiqui J.A., Maurya S.K., Lakshmanan I., Jain M., Ganti A.K., Salgia R., Batra S.K., Nasser M.W. (2020). Epigenetic landscape of small cell lung cancer: Small image of a giant recalcitrant disease. Semin. Cancer Biol..

[27] Nakagawa M., Takizawa N., Narita M., Ichisaka T., Yamanaka S. (2010). Promotion of direct reprogramming by transformation-deficient Myc. Proc. Natl. Acad. Sci. USA.

[28] Schaefer T., Lengerke C. (2020). SOX2 protein biochemistry in stemness, reprogramming, and cancer: The PI3K/AKT/SOX2 axis and beyond. Oncogene.

[29] Beltran H., Demichelis F. (2021). Therapy considerations in neuroendocrine prostate cancer: What next?. Endocr.-Relat. Cancer.

[30] Mosquera J.M., Beltran H., Park K., MacDonald T.Y., Robinson B.D., Tagawa S.T., Perner S., Bismar T.A., Erbersdobler A., Dhir R. (2013). Concurrent AURKA and MYCN gene amplifications are harbingers of lethal treatmentrelated neuroendocrine prostate cancer. Neoplasia.

[31] Hobeika C., Rached G., Eid R., Haddad F., Chucri S., Kourie H.R., Kattan J. (2018). ALK-rearranged adenocarcinoma transformed to small-cell lung cancer: A new entity with specific prognosis and treatment?. Pers. Med..

[32] Calabrese F., Pezzuto F., Lunardi F., Fortarezza F., Tzorakoleftheraki S.-E., Resi M.V., Tiné M., Pasello G., Hofman P. (2022). Morphologic-Molecular Transformation of Oncogene Addicted Non-Small Cell Lung Cancer. Int. J. Mol. Sci..

[33] Schneider J.L., Lin J.J., Shaw A.T. (2023). ALK-positive lung cancer: A moving target. Nat. Cancer.

[34] Wu J., Lin Z. (2022). Non-Small Cell Lung Cancer Targeted Therapy: Drugs and Mechanisms of Drug Resistance. Int. J. Mol. Sci..

[35] Fujita S., Masago K., Katakami N., Yatabe Y. (2016). Transformation to SCLC after treatment with the ALK inhibitor alectinib. J. Thorac. Oncol..

[36] Miyamoto S., Ikushima S., Ono R., Awano N., Kondo K., Furuhata Y., Fukumoto K., Kumasaka T. (2016). Transformation to small-cell lung cancer as a mechanism of acquired resistance to crizotinib and alectinib. Jpn. J. Clin. Oncol..

[37] Yamagata A., Yokoyama T., Fukuda Y., Ishida T. (2021). Alectinib re-challenge in small cell lung cancer transformation after chemotherapy failure in a patient with ALK-positive lung cancer: A case report. Respir. Med. Case Rep..

[38] Shaw A., Riely G., Bang Y.-J., Kim D.-W., Camidge D., Solomon B., Varella-Garcia M., Iafrate A., Shapiro G., Usari T. (2019). Crizotinib in ROS1-rearranged advanced non-small-cell lung cancer (NSCLC): Updated results, including overall survival, from PROFILE 1001. Ann. Oncol..

[39] Drilon A., Jenkins C., Iyer S., Schoenfeld A., Keddy C., Davare M.A. (2021). ROS1-dependent cancers—Biology, diagnostics and therapeutics. Nat. Rev. Clin. Oncol..

[40] Drilon A., Siena S., Dziadziuszko R., Barlesi F., Krebs M.G., Shaw A.T., de Braud F., Rolfo C., Ahn M.-J., Wolf J. (2020). Entrectinib in ROS1 fusion-positive non-small-cell lung cancer: Integrated analysis of three phase 1–2 trials. Lancet Oncol..

[41] Cho B.C., Drilon A.E., Doebele R.C., Kim D.-W., Lin J.J., Lee J., Ahn M.-J., Zhu V.W., Ejadi S., Camidge D.R. (2019). Safety and preliminary clinical activity of repotrectinib in patients with advanced ROS1 fusion-positive non-small cell lung cancer (TRIDENT-1 study). J. Clin. Oncol..

[42] Gainor J.F., Tseng D., Yoda S., Dagogo-Jack I., Friboulet L., Lin J.J., Hubbeling H.G., Dardaei L., Farago A.F., Schultz K.R. (2017). Patterns of Metastatic Spread and Mechanisms of Resistance to Crizotinib in ROS1-Positive Non–Small-Cell Lung Cancer. JCO Precis. Oncol..

[43] Shaw A.T., Solomon B.J., Chiari R., Riely G.J., Besse B., Soo R.A., Kao S., Lin C.-C., Bauer T.M., Clancy J.S. (2019). Lorlatinib in advanced ROS1-positive non-small-cell lung cancer: A multicentre, open-label, single-arm, phase 1–2 trial. Lancet Oncol..

[44] García-Pardo M., Calles A. (2021). ROS-1 NSCLC therapy resistance mechanism. Precis. Cancer Med..

[45] Filetti M., Piras M., Giusti R. (2021). Profilazione genomica completa nel carcinoma polmonare neuroendocrino a grandi cellule: I tempi stanno cambiando [Comprehensive genomic profiling in large cell neuroendocrine lung cancer: The times they are a-changin]. Recent. Prog. Med..

[46] Ko H.-J., Hsu C.-K., Yeh Y.-C., Huang H.-C. (2022). ROS-1 TKI for the treatment of concurrent sarcomatoid transformation and acquired ROS-1 F2004C mutation in a lung adenocarcinoma patient. Pulmonology.

[47] Poulin E.J., Bera A.K., Lu J., Lin Y.-J., Strasser S.D., Paulo J.A., Huang T.Q., Morales C., Yan W., Cook J. (2019). Tissue-Specific Oncogenic Activity of KRASA146T. Cancer Discov..

[48] Koga T., Suda K., Fujino T., Ohara S., Hamada A., Nishino M., Chiba M., Shimoji M., Takemoto T., Arita T. (2021). KRAS Secondary Mutations That Confer Acquired Resistance to KRAS G12C Inhibitors, Sotorasib and Adagrasib, and Overcoming Strategies: Insights From In Vitro Experiments. J. Thorac. Oncol..

[49] Hallin J., Engstrom L.D., Hargis L., Calinisan A., Aranda R., Briere D.M., Sudhakar N., Bowcut V., Baer B.R., Ballard J.A. (2020). The KRASG12C Inhibitor MRTX849 Provides Insight toward Therapeutic Susceptibility of KRAS-Mutant Cancers in Mouse Models and Patients. Cancer Discov..

[50] Hillig R.C., Sautier B., Schroeder J., Moosmayer D., Hilpmann A., Stegmann C.M., Werbeck N.D., Briem H., Boemer U., Weiske J. (2019). Discovery of potent SOS1 inhibitors that block RAS activation via disruption of the RAS–SOS1 interaction. Proc. Natl. Acad. Sci. USA.

[51] Xue J., Zhao Y., Aronowitz J., Mai T.T., Vides A., Qeriqi B., Kim D., Li C., De Stanchina E., Mazutis L. (2020). Rapid non-uniform adaptation to conformation-specific KRAS(G12C) inhibition. Nature.

[52] Misale S., Fatherree J.P., Cortez E., Li C., Bilton S.J., Timonina D., Myers D.T., Lee D., Gomez-Caraballo M., Greenberg M. (2019). KRAS G12C NSCLC Models Are Sensitive to Direct Targeting of KRAS in Combination with PI3K Inhibition. Clin. Cancer Res..

[53] Li A.Y., McCusker M.G., Russo A., Scilla K.A., Gittens A., Arensmeyer K., Mehra R., Adamo V., Rolfo C. (2019). RET fusions in solid tumors. Cancer Treat. Rev..

[54] Drilon A., Oxnard G.R., Tan D.S., Loong H.H., Johnson M., Gainor J., McCoach C.E., Gautschi O., Besse B., Cho B.C. (2020). Efficacy of Selpercatinib in RET Fusion–Positive Non–Small-Cell Lung Cancer. N. Engl. J. Med..

[55] Lin J., Liu S., McCoach C., Zhu V., Tan A., Yoda S., Peterson J., Do A., Prutisto-Chang K., Dagogo-Jack I. (2020). Mechanisms of resistance to selective RET tyrosine kinase inhibitors in RET fusion-positive non-small-cell lung cancer. Ann. Oncol..

[56] Gainor J.F., Curigliano G., Kim D.-W., Lee D.H., Besse B., Baik C.S., Doebele R.C., Cassier P.A., Lopes G., Tan D.S.W. (2021). Pralsetinib for RET fusion-positive non-small-cell lung cancer (ARROW): A multi-cohort, open-label, phase 1/2 study. Lancet Oncol..

[57] Pishdad R., Illei P.B., Gocke C.D., Ball D.W. (2024). RET gene fusion and emergent Selpercatinib resistance in a calcitonin-rich neuroendocrine carcinoma: A case report. Front. Oncol..

[58] Ettinger D.S., Wood D.E., Aisner D.L., Akerley W., Bauman J.R., Bharat A., Bruno D.S., Chang J.Y., Chirieac L.R., D’amico T.A. (2021). NCCN Guidelines Insights: Non–Small Cell Lung Cancer, Version 2.2021. J. Natl. Compr. Cancer Netw..

[59] Recondo G., Bahcall M., Spurr L., Che J., Ricciuti B., Leonardi G.C., Lo Y.-C., Li Y.Y., Lamberti G., Nguyen T. (2020). Molecular Mechanisms of Acquired Resistance to MET Tyrosine Kinase Inhibitors in Patients with MET Exon 14–Mutant NSCLC. Clin. Cancer Res..

[60] Leonetti A., Facchinetti F., Rossi G., Minari R., Conti A., Friboulet L., Tiseo M., Planchard D. (2018). BRAF in non-small cell lung cancer (NSCLC): Pickaxing another brick in the wall. Cancer Treat. Rev..

[61] Niemantsverdriet M., Schuuring E., Ter Elst A., van der Wekken A.J., van Kempen L.C., Berg A.v.D., Groen H.J. (2018). KRAS Mutation as a Resistance Mechanism to BRAF/MEK Inhibition in NSCLC. J. Thorac. Oncol..

[62] Abravanel D.L., Nishino M., Sholl L.M., Ambrogio C., Awad M.M. (2018). An Acquired NRAS Q61K Mutation in BRAF V600E-Mutant Lung Adenocarcinoma Resistant to Dabrafenib Plus Trametinib. J. Thorac. Oncol..

[63] Nana F.A., Ocak S. (2021). Targeting BRAF Activation as Acquired Resistance Mechanism to EGFR Tyrosine Kinase Inhibitors in EGFR-Mutant Non-Small-Cell Lung Cancer. Pharmaceutics.

[64] Mariniello A., Righi L., Morrone A., Carnio S., Bironzo P. (2022). Squamous cell histological transformation in a lung adenocarcinoma patient (hyper) progressing upon immunotherapy. Tumori J..

[65] Solomon J.P., Benayed R., Hechtman J.F., Ladanyi M. (2019). Identifying patients with NTRK fusion cancer. Ann. Oncol..

[66] Drilon A., Laetsch T.W., Kummar S., Dubois S.G., Lassen U.N., Demetri G.D., Nathenson M., Doebele R.C., Farago A.F., Pappo A.S. (2018). Efficacy of Larotrectinib in TRK Fusion–Positive Cancers in Adults and Children. N. Engl. J. Med..

[67] Imakita T., Fujita K., Kanai O., Okamura M., Hashimoto M., Nakatani K., Sawai S., Mio T. (2021). Small cell transformation of non-small cell lung cancer under immunotherapy: Case series and literature review. Thorac. Cancer.

[68] Bar J., Ofek E., Barshack I., Gottfried T., Zadok O., Kamer I., Urban D., Perelman M., Onn A. (2019). Transformation to small cell lung cancer as a mechanism of resistance to immunotherapy in non-small cell lung cancer. Lung Cancer.

[69] Du F., Han Y., Hu X., Xiao Y., Shi Y., Sun J., Sun Z., Yang Y., Yu J., Zhang X. (2023). Large cell neuroendocrine carcinoma transformation: A novel acquired drug resistance mechanism in colorectal adenocarcinoma. Cancer Innov..

[70] Xin G., Song N., Jiang K. (2024). Esophageal squamous cell carcinoma transformed into neuroendocrine carcinoma after neoadjuvant immunochemotherapy: A case report. Oncol. Lett..

[71] Sehgal K., Varkaris A., Viray H., VanderLaan P.A., Rangachari D., Costa D.B. (2020). Small cell transformation of non-small cell lung cancer on immune checkpoint inhibitors: Uncommon or under-recognized?. J. Immunother. Cancer.

[72] Gettinger S.N., Wurtz A., Goldberg S.B., Rimm D., Schalper K., Kaech S., Kavathas P., Chiang A., Lilenbaum R., Zelterman D. (2018). Clinical features and management of acquired resistance to PD-1 axis inhibitors in 26 patients with advanced non–small cell lung cancer. J. Thorac. Oncol..

[73] Roca E., Gurizzan C., Amoroso V., Vermi W., Ferrari V., Berruti A. (2017). Outcome of patients with lung adenocarcinoma with transformation to small-cell lung cancer following tyrosine kinase inhibitors treatment: A systematic review and pooled analysis. Cancer Treat. Rev..

[74] Liu Y. (2018). Small cell lung cancer transformation from EGFR-mutated lung adenocarcinoma: A case report and literatures review. Cancer Biol. Ther..

[75] Zhang Y., Tang Y., Xu Y., Guo W.-H., Li Y.-C., Liu X.-K., Huang C.-Y., Wang Y.-S., Wei Y.-Q. (2013). Rapid increase of serum neuron specific enolase level and tachyphylaxis of EGFR-tyrosine kinase inhibitor indicate small cell lung cancer transformation from EGFR positive lung adenocarcinoma?. Lung Cancer.

[76] Xie T., Li Y., Ying J., Cai W., Li J., Lee K.Y., Ricciuti B., Pacheco J., Xing P. (2020). Whole exome sequencing (WES) analysis of transformed small cell lung cancer (SCLC) from lung adenocarcinoma (LUAD). Transl. Lung Cancer Res..

[77] Rudin C.M., Brambilla E., Faivre-Finn C., Sage J. (2021). Small-cell lung cancer. Nat. Rev. Dis. Prim..

[78] Leonetti A., Minari R., Mazzaschi G., Gnetti L., La Monica S., Alfieri R., Campanini N., Verzè M., Olivani A., Ventura L. (2021). Small cell lung cancer transformation as a resistance mechanism to osimertinib in epidermal growth factor receptor-mutated lung adenocarcinoma: Case report and literature review. Front. Oncol..

[79] Inoue-Yamauchi A., Jeng P.S., Kim K., Chen H.-C., Han S., Ganesan Y.T., Ishizawa K., Jebiwott S., Dong Y., Pietanza M.C. (2017). Targeting the differential addiction to anti-apoptotic BCL-2 family for cancer therapy. Nat. Commun..

[80] Eastman S.S.R.A. (2016). BCL2 inhibitors as anticancer drugs: A plethora of misleading bh3 mimetics. Mol. Cancer Ther..

[81] Witkiewicz A.K., Chung S., Brough R., Vail P., Franco J., Lord C.J., Knudsen E.S. (2018). Targeting the vulnerability of rb tumor suppressor loss in triple-negative Breast Cancer. Cell Rep..

[82] Liu J.C., Granieri L., Shrestha M., Wang D.-Y., Vorobieva I., Rubie E.A., Jones R., Ju Y., Pellecchia G., Jiang Z. (2018). Identification of CDC25 as a Common Therapeutic Target for Triple-Negative Breast Cancer. Cell Rep..

[83] Shah K.N., Bhatt R., Rotow J., Rohrberg J., Olivas V., Wang V.E., Hemmati G., Martins M.M., Maynard A., Kuhn J. (2019). Aurora kinase A drives the evolution of resistance to third-generation EGFR inhibitors in lung cancer. Nat. Med..

[84] Horn L., Mansfield A.S., Szczęsna A., Havel L., Krzakowski M., Hochmair M.J., Huemer F., Losonczy G., Johnson M.L., Nishio M. (2018). First-line atezolizumab plus chemotherapy in extensive-stage small-cell lung cancer. N. Engl. J. Med..

[85] Tokaca N., Wotherspoon A., Nicholson A.G., Fotiadis N., Thompson L., Popat S. (2017). Lack of response to nivolumab in a patient with EGFR -mutant non-small cell lung cancer adenocarcinoma sub-type transformed to small cell lung cancer. Lung Cancer.

[86] Nishikawa S., Tambo Y., Ninomiya H., Oguri T., Kawashima Y., Takano N., Kitazono S., Ohyanagi F., Horiike A., Yanagitani N. (2016). A case treated with nivolumab after small cell lung cancer transformation of mutant EGFR non-small cell lung cancer. Ann. Oncol..

[87] Wang S., Xie T., Hao X., Wang Y., Hu X., Wang L., Li Y., Li J., Xing P. (2021). Comprehensive analysis of treatment modes and clinical outcomes of small cell lung cancer transformed from epidermal growth factor receptor mutant lung adenocarcinoma. Thorac. Cancer.

[88] Ding J., Leng Z., Gu H., Jing X., Song Y. (2023). Etoposide/platinum plus anlotinib for patients with transformed small-cell lung cancer from EGFR-mutant lung adenocarcinoma after EGFR-TKI resistance: A retrospective and observational study. Front. Oncol..

