# Supplementary material for: Transformation to Neuroendocrine Phenotype in Non-Small-Cell Lung Carcinoma: A Literature Review
Source: Int J Mol Sci. 2025 May 26;26(11):5096. doi: 10.3390/ijms26115096 (PMC12154276; doi:10.3390/ijms26115096)
Supplement: Supplementary file 1 [file ijms-26-05096-s001.zip › ijms-3591833-supplementary.pdf]

Table S1. Glossary of Abbreviations Used Throughout the Manuscript

Abbreviation	Full Term
NSCLC	Non-Small Cell Lung Cancer
SCLC	Small Cell Lung Cancer
LUAD	Lung Adenocarcinoma
LUSC	Lung Squamous Cell Carcinoma
EGFR	Epidermal Growth Factor Receptor
TKI	Tyrosine Kinase Inhibitor
CT	Chemotherapy
IO	Immunotherapy
PD-1	Programmed Cell Death Protein 1
PD-L1	Programmed Death-Ligand 1
NSE	Neuron-Specific Enolase
pro-GRP	Pro-Gastrin-Releasing Peptide
NGS	Next Generation Sequencing
PI3K	Phosphatidylinositol 3-Kinase
AKT	AKT serine/threonine kinase
PTEN	Phosphatase and Tensin Homolog
NOTCH	Neurogenic locus notch homolog
ASCL1	Achaete-scute homolog 1
MYC	Myelocytomatosis oncogene
SOX2	SRY-Box Transcription Factor 2
AURKA	Aurora Kinase A
RB1	Retinoblastoma 1
TP53	Tumor Protein P53
RET	Rearranged during Transfection
ALK	Anaplastic Lymphoma Kinase
ROS1	c-ros oncogene 1
KRAS	Kirsten Rat Sarcoma Viral Oncogene
BRAF	B-Raf Proto-Oncogene
NTRK	Neurotrophic Tyrosine Receptor Kinase
FGFR	Fibroblast Growth Factor Receptor
CDK5	Cyclin-dependent kinase 5
CDK4/6	Cyclin-dependent kinases 4 and 6

CHK	Checkpoint Kinase
PLK	Polo-like Kinase
EZH2	Enhancer of Zeste Homolog 2
MAP2K1	Mitogen-activated protein kinase kinase 1

Table S2. Inhibitors And Their Targeted Mutations

**Inhibitor**	**Targeted Mutation(s)**
Osimertinib	EGFR exon 19 del, L858R, T790M
Afatinib	EGFR exon 19 del, L858R
Gefitinib	EGFR exon 19 del, L858R
Erlotinib	EGFR exon 19 del, L858R
Dacomitinib	EGFR exon 19 del, L858R
Sotorasib	KRAS G12C
Adagrasib	KRAS G12C
Alectinib	ALK fusions
Brigatinib	ALK fusions
Lorlatinib	ALK fusions, ALK G1202R
Crizotinib	ALK, ROS1 fusions, MET
Ceritinib	ALK fusions
Entrectinib	ROS1, NTRK fusions
Repotrectinib	ROS1 G2032R, NTRK fusions
Selpercatinib	RET fusions
Pralsetinib	RET fusions
Capmatinib	MET exon 14 skipping
Tepotinib	MET exon 14 skipping
Dabrafenib + Trametinib	BRAF V600E
Larotrectinib	NTRK fusions
